# The Regioselective Conjugation of the 15-nt Thrombin Aptamer with an Optimized Tripeptide Sequence Greatly Increases the Anticoagulant Activity of the Aptamer

**DOI:** 10.3390/pharmaceutics15020604

**Published:** 2023-02-10

**Authors:** Irina V. Varizhuk, Vladimir B. Tsvetkov, Ilya Yu. Toropygin, Andrey A. Stomakhin, Natalia A. Kolganova, Sergei A. Surzhikov, Edward N. Timofeev

**Affiliations:** 1 Engelhardt Institute of Molecular Biology, Russian Academy of Sciences, 119991 Moscow, Russia; 2 Federal Research and Clinical Center of Physical-Chemical Medicine, 119435 Moscow, Russia; 3 Institute of Biodesign and Complex System Modeling, Sechenov First Moscow State Medical University, 119146 Moscow, Russia; 4 Department of Proteomics, V.N. Orekhovich Research Institute of Biomedical Chemistry, Russian Academy of Medical Sciences, Moscow 119832, Russia

**Keywords:** therapeutic oligonucleotide, thrombin aptamer, peptide conjugate, anticoagulant, G-quadruplex

## Abstract

Currently, oligonucleotide therapy has emerged as a new paradigm in the treatment of human diseases. In many cases, however, therapeutic oligonucleotides cannot be used directly without modification. Chemical modification or the conjugation of therapeutic oligonucleotides is required to increase their stability or specificity, improve their affinity or inhibitory characteristics, and address delivery issues. Recently, we proposed a conjugation strategy for a 15-nt G-quadruplex thrombin aptamer aimed at extending the recognition interface of the aptamer. In particular, we have prepared a series of designer peptide conjugates of the thrombin aptamer, showing improved anticoagulant activity. Herein, we report a new series of aptamer–peptide conjugates with optimized peptide sequences. The anti-thrombotic activity of aptamer conjugates was notably improved. The lead conjugate, TBA–GLE, was able to inhibit thrombin-induced coagulation approximately six-fold more efficiently than the unmodified aptamer. In terms of its anticoagulant activity, the TBA–GLE conjugate approaches NU172, one of the most potent G-quadruplex thrombin aptamers. Molecular dynamics studies have confirmed that the principles applied to the design of the peptide side chain are efficient instruments for improving aptamer characteristics for the proposed TBA conjugate model.

## 1. Introduction

The development of oligonucleotide-based therapeutics has shown considerable progress in the recent years [1]. Permanent interest in oligonucleotides as therapeutic agents has arisen from multiple factors that include a wide range of druggable targets, well-established and relatively inexpensive methods of oligonucleotide preparation and modification, a range of molecular mechanisms of their therapeutic action available for selection, new emerging methods of delivery, and a high specificity of targeting [2,3,4,5]. Currently, the most frequently used types of therapeutic oligonucleotides are antisense oligonucleotides, small interfering RNAs, gene-editing guide RNAs, and aptamers. The latter represent quite an unusual class of the oligonucleotide therapeutic agents as they recruit base complementarity to form a unique secondary structure, and bind their targets with high specificity using all available arsenals of molecular recognition [4,6]. Due to a virtually unlimited range of targeted molecules, aptamers may function as therapeutics, diagnostic probes, or even delivery vehicles for other agents. In this respect, the development of aptamer conjugates is a highly in demand field of study as a way to improve the affinity or functional characteristics of aptamers [3,7,8]. Depending on the functional roles of subunits in the oligonucleotide conjugate, this may include lipids, carbohydrates, peptides, or some other functional elements [9,10]. Particularly, aptamer–peptide conjugates may exhibit improved precision of targeting, enhanced affinity [11,12,13], or better delivery [10,11] as compared to unmodified aptamers or unconjugated peptides.

**Figure 1:**
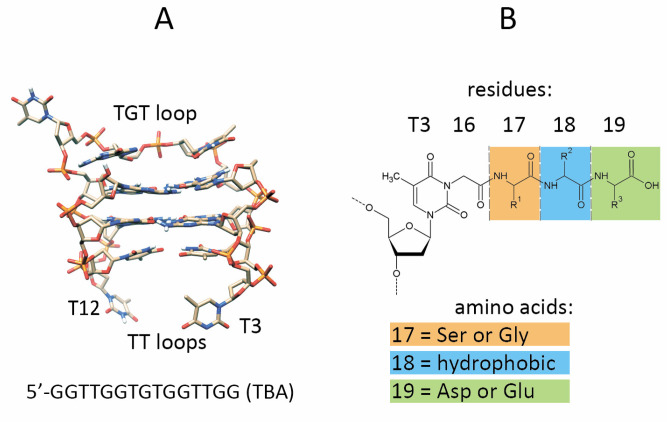
Structure of the 15-nt thrombin aptamer (TBA). Two-layer anti-parallel G-quadruplex is formed by a single DNA strand and includes two TT loops and one TGT loop. (**A**) Design of the tripeptide subunit in TBA conjugates. The peptide side chain is attached to the thymine base through an N3-carboxymethyl linker (residue 16) (**B**).

Recently, we reported the preparation of thrombin binding aptamer (TBA)—peptide conjugates with improved anticoagulant characteristics [13]. A TBA is a 15-nt G-rich DNA sequence 5′-GGTTGGTGTGGTTGG that forms a two-shelf anti-parallel G-quadruplex and binds with high affinity thrombin at its exosite I (Figure 1A). In the previous study, the tripeptide sequences were selected on the basis of general considerations regarding the amino-acid composition at the protein–protein interfaces. The anticoagulant activities of the conjugates appeared to be either improved or decreased depending on the particular peptide sequence context. Nevertheless, we have found that, in a series of five TBA–peptide conjugates bearing tripeptide side chains at T3 residue, two variants exhibited considerably improved anticoagulant properties. A further analysis of the thrombin complexes with TBA conjugates by molecular dynamics revealed specific requirements for each of the three amino acid residues in the peptide subunit. In particular, amino acids proximal to the aptamer (residue 17) should not be aromatic like tryptophan or tyrosine to avoid intramolecular π–π stacking interactions with the T3 thymine nucleobase. Furthermore, amino acid 18 in the middle of the peptide subunit is expected to be hydrophobic or aromatic to optimize interactions with Leu65, Ile82, or Met84 of the thrombin. Finally, terminal amino acid 19 at the C-end of the peptide should preferably bear two carboxyl groups due to the presence of lysine residues 109 and 110 on the thrombin surface nearby. To verify these rules as a guide in the rational design of efficient TBA–peptide conjugates, we prepared a new series of TBA–peptide conjugates (Figure 1B) and studied their biophysical and inhibitory characteristics. In the current study, the new design of the tripeptide subunit satisfies the above rules for selection. Here, we show that the optimized sequence of the peptide subunit boosts the anticoagulant activity of the aptamer conjugates far beyond the majority of known 15-nt TBA analogues.

## 2. Materials and Methods

### 2.1. Materials

Chemical reagents and solvents were purchased from various commercial suppliers and used without further purification. Modified thymidine phosphoramidite (Figure S1) was prepared as previously described [13]. Research-grade human thrombin from plasma for clotting studies was purchased from Renam (Moscow, Russia; #P33). Fibrinogen from human plasma was obtained from Sigma-Aldrich (St. Louis, MO, USA; #F3879). Standard reagents for automated oligonucleotide synthesis were purchased from Glen Research (Sterling, VA, USA; #10-1020, #10-1030, #20-2010). Synthetic tripeptides were purchased from Cloud-Clone Corp. (Wuhan, China).

### 2.2. Automated Oligonucleotide Synthesis and Conjugation

DNA oligomers were synthesized using an ABI 3400 DNA/RNA synthesizer in DMTr-on mode. The coupling time for modified thymidine phosphoramidite was set to 60 s. TBA–peptide conjugates were prepared by reacting the support-bound protected modified TBA sequence with the respective peptide (10 mg) in a mixture of 0.2 M sodium carbonate (pH 9.5) and DMF (2:1, *v*/*v*, 150 μL) for 24 h at 4 °C. Partial deprotection of the conjugates was carried out with concentrated ammonia for 6 h at 55 °C. DMTr-protected oligonucleotides were purified by reverse-phase HPLC (Hypersil ODS, 5 μm, 4.6 × 250 mm; 10–50% MeCN in 50 mM TEAA for 30 min). After removal of the 5′ DMTr group, oligomers were repeatedly purified by reverse-phase HPLC (0–25% MeCN in 50 mM TEAA for 30 min), with the control of the fractions by MALDI mass spectrometry and denaturing gel electrophoresis. Additional purification was carried out by preparative electrophoresis in denaturing 20% polyacrylamide gel (9:1) containing 7 M urea. Variant TBA-SPE contained approximately 25% of unconjugated full-length oligomer. The TBA–peptide conjugate concentrations were determined by measuring the UV absorbance at 260 nm. The molar extinction coefficients for TBA–peptide conjugates were calculated using previously reported extinction coefficients for tryptophan and tyrosine at 260 nm (3787 M^−1^ cm^−1^ and 582 M^−1^ cm^−1^, respectively) [14].

### 2.3. MALDI Mass Spectrometry

The mass spectra of TBA conjugates were acquired using an Ultraflex MALDI-TOF/TOF mass spectrometer (Bruker Daltonics, Billerica, USA) and a 4800 Plus MALDI-TOF mass spectrometer (AB Sciex, Framingham, MA, USA) in the linear mode. The analysis was performed using 0.25 M aqueous 3-hydroxypicolinic acid as the matrix and 10 mM diammonium citrate as an additive. Spectra were recorded for positive ions. Before analysis, the samples were treated with Dowex 50WX8 in ammonium form.

### 2.4. Ultraviolet Thermal Denaturation

The absorbance versus temperature profiles were obtained with a Cary 3500 UV–VIS spectrophotometer equipped with a Peltier cell holder (Agilent Technologies, Santa Clara, CA, USA). Melting experiments were performed at 295 nm in 10 mM sodium cacodylate (pH 7.4) and 100 mM KCl. The heating rate was 0.5 °C/min. The melting curves were fitted to a two-state intramolecular model with equal baseline slopes. The melting temperatures were calculated using ΔH and ΔS values. The oligonucleotide concentration was 5 μM.

### 2.5. Circular Dichroism Spectroscopy

Circular dichroism (CD) measurements were performed at 25 °C with an aptamer concentration of 5 μM in 10 mM sodium cacodylate (pH 7.4) and 100 mM KCl using a Jasco-715 CD spectrometer (JASCO, Easton, MD, USA). The spectra were obtained at a bandwidth of 1 nm.

### 2.6. Fibrinogen Clotting in the Presence of Aptamers

Human thrombin (50 μL, 10 U/mL) was added to a solution of fibrinogen (2 mg/mL) and aptamer (30 nM) in 1 mL of PBS in a quartz cuvette in a temperature-controlled cuvette holder of a spectrophotometer at 25 °C. Monitoring of the absorbance at 360 nm started immediately after the addition of thrombin and was stopped after the curve reached a plateau. The measurements were carried out in several experimental series. Each series included a blank sample (without aptamer) and TBA control. The clotting curve for each sample was measured at least in duplicate.

### 2.7. Molecular Dynamics Simulations of TBA–Peptide Complexes with Thrombin

Molecular dynamics studies were performed as described previously [13]. Briefly, the crystal structure of the thrombin complex with T3-modified TBA (PDB id 6Z8X) was used as a starting model to build TBA–peptide conjugates. The arrangement of the loops matched the same in the crystal structure PDB id 1HAO. Optimized tripeptide subunits were added to the terminal glycine at T3. The conjugate models were optimized by the SYBYL X and Powell method.

The TBA–peptide model stabilities were verified by MD simulations using Amber 20 software [15]. The influence of the solvent was simulated using the OPC3 water model [16]. Rectangular box and periodic boundary conditions were used in the simulation. The space between the TBA OPC model and the periodic box wall was at least 15 Å. Potassium ions were used to neutralize the negative charge of the DNA backbone and stabilize the quadruplex structure. The parameters needed for interatomic energy calculation were taken from the force fields OL15 [17,18] for the DNA and ff14SBonlysc [19] for the peptides. 

After the two-stage optimization procedure [13], TBA–peptide complexes with thrombin were run in MD simulations using constant temperature (T = 300 K) and constant pressure (*p* = 1 atm) for 80 ns. The plots of energy of interaction vs. time were smoothed using the moving average method (span = 5).

## 3. Results and Discussion

### 3.1. Design and Synthesis of TBA–Peptide Conjugates

As mentioned above, the optimized design of the peptide subunit suggests a non-aromatic and probably non-hydrophobic amino acid in position 17 (Figure 1B). Therefore, we excluded aromatic and hydrophobic amino acids from the selection at position 17. We reasoned that a methyl or hydroxymethyl group in Gly or Ser would have a minimal effect on possible intramolecular interactions with the thymine base or other groups of the oligonucleotide. Additionally, in order to limit the total number of possible conjugate variants, we used only two amino acid residues, glycine or serine, at the N-terminus of the peptide. The selection of the residue at the C-terminus was facilitated by the fact that only two natural amino acids satisfy the above rules, i.e., aspartic and glutamic acids. Since we used asparagine as a C-terminal non-aromatic residue in the peptide subunits in our previous study, we included a single tripeptide Ser-Tyr-Asn (SYN) conjugate into new conjugate series for comparative purposes. In the tripeptide side chain, amino acid 18 is assumed to be the most important to support an extended recognition interface through interactions with the protein residues Met84, Leu65, or Ile82. Due to the hydrophobic nature of these protein residues, we presumed that amino acids Leu, Ile, Val, Pro, Tyr, and Trp are the best candidates to examine position 18 of the peptide subunit. In total, ten TBA conjugates were designed as variants of the optimized set (Table 1).

The synthesis of the peptide conjugates was carried out using a modified thymidine phosphoramidite synthetic block (Figure S1). The preparation of modified phosphoramidite and peptide conjugates has previously been described [13]. Briefly, 5′-O-(4,4′-dimethoxytrityl)-thymidine was converted to 5′-O-(4,4′-dimethoxytrityl)-N3-carboxymethylthymidine triethylammonium salt by the reaction with ethyl bromoacetate in the presence of 1,8-diazabicyclo [5.4.0]undec-7-ene followed by alkaline hydrolysis. The carboxylic group was further activated with p-nitrophenyl trifluoroacetate to yield 5′-O-(4,4′-dimethoxytrityl)-N3-(p-nitrophenoxycarbonylmethyl)-thymidine. The latter compound was then converted to the respective phosphoramidite. The phosphoramidite building block was further utilized for the synthesis of a TBA oligonucleotide with an activated carboxylic function at T3. After the oligonucleotide synthesis, a solid support with an anchored p-nitrophenyl activated TBA sequence reacted with the respective tripeptide in a sodium carbonate buffer (pH 9.5) at 4 °C for 24 h. Complete oligonucleotide deprotection was achieved by the standard ammonia treatment at 55 °C.

Due to limited efficiency of conjugation and due to the partial hydrolysis of activated oligonucleotide, the crude product contained a complex mixture of the peptide conjugate, oligomer with unreacted side chain amide, oligomer with unreacted side chain carboxylic acid, and failure sequences. Depending on the amino acid composition in the peptide subunit, the retention times of the conjugates in reversed phase HPLC or their migration rates in polyacrylamide gel electrophoresis varied. Therefore, the combination of the two separation techniques was typically required for the efficient purification of the TBA–peptide conjugates (Figures S2 and S3).

### 3.2. Biophysical Characteristics of TBA–Peptide Conjugates

**Figure 2:**
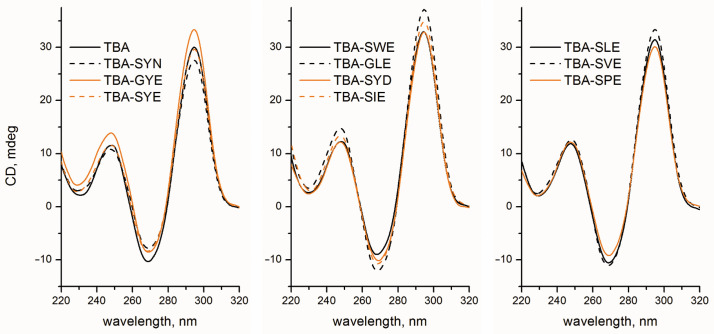
CD spectra of TBA–peptide conjugates at 25 °C with a conjugate concentration of 5 μM in 10 mM sodium cacodylate (pH 7.4) and 100 mM KCl. All conjugates show typical anti-parallel G-quadruplex fold.

Similar to a previous series of TBA conjugates [13], all new peptide–oligonucleotide chimeras were able to reproduce an antiparallel two-layer G-quadruplex fold typical for the parent TBA. Regardless of the peptide subunit, the CD spectra of conjugates in 100 mM KCl buffer exhibited a negative band at 268 nm and two positive bands at 248 and 293 nm (Figure 2). Variations in band intensities for the different conjugate variants may be associated with an insignificant effect of a peptide side chain on the quadruplex architecture.

The melting temperature measurements (Table 1) show the absence of notable interactions between the tripeptide unit and the G-quadruplex module which might affect the thermal stability of the conjugates. The stability of the TBA conjugates with optimized tripeptide sequences in the 100 mM KCl buffer was nearly identical to that of the unmodified aptamer. An increase in the *T*_m_ value did not exceed 1 °C for the majority of the entries in Table 1. This observation is in notable contrast with the results of a previous study showing Δ*T*_m_ up to 5.4 °C (for TBA-FYW) [13], which was associated with the aromatic stacking of the T3 pyrimidine base. Thus, the rational optimization of the peptide sequences appeared successful in preventing intramolecular stacking interactions between the T3 nucleobase and aromatic residues of the tripeptides.

### 3.3. Anticoagulant Properties of TBA–Peptide Conjugates

**Figure 3:**
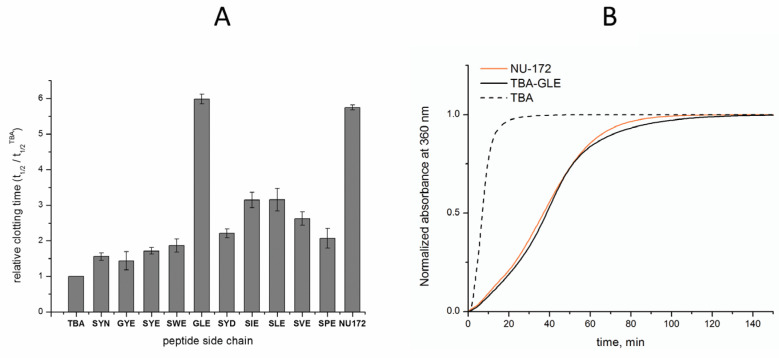
Graph of anticoagulant activity of TBA–peptide conjugates (**A**). Normalized fibrinogen clotting curves in PBS at 25 °C in the presence of TBA, TBA–GLE, or NU172 (**B**).

The examination of the inhibitory characteristics of TBA–peptide conjugates was performed in fibrinogen clotting time tests [21]. The polymerization of fibrinogen in the presence of thrombin and TBA conjugates was monitored by the increase in the absorbance of samples at 360 nm due to light scattering. Since we observed certain variations in the clotting time of the control samples (TBA and blank samples) in different experimental series, we used the relative clotting time values, defined as a ratio t_1/2_/t_1/2_^TBA^, in which t_1/2_ is the time required to reach 50% of the absorbance maximum. The results of the coagulation tests are presented in Table 1 and Figure 3. None of tested samples showed a clotting time lower than that in the TBA control. The conjugate TBA-SYN, which was designed to extend a previously studied series of conjugates, showed a relative value of 1.56, thus exceeding the highest results of the previous study (1.38) [13]. A comparative analysis of particular amino acid residues at specific positions in terms of their effect on anticoagulant activities suggests that glycine is probably a more appropriate residue than serine at position 17. Although TBA–SYE is somewhat more active than TBA-GYE, the striking difference between the data for the GLE and SLE conjugates strongly supports this point of view. A comparison of the clotting times for the conjugates with different amino acid residues in the middle of the peptide clearly points to the advantages of non-aromatic hydrophobic groups in this position over aromatic groups. This finding is supported by the direct comparison of the homologous peptide side chains: GLE vs. GYE; SIE, SLE, and SVE vs. SYE, SPE, and SWE. When considering C-terminal amino acids, we assume that aspartic acid is somewhat more advantageous than glutamic acid, according to the data for TBA–SYD and TBA–SYE. The relative anticoagulant efficiency for conjugates with the peptide sequences GLE, SYD, SIE, SLE, SVE, and SPE exceeded the threshold of 2. The most prolonged clotting time was observed for the TBA–GLE conjugate. A t_1/2_/t_1/2_^TBA^ value of 5.98 was found for this conjugate. To provide direct reference to the most advanced G-quadruplex aptamer anticoagulants, we included into our study NU172, a highly potent bimodular duplex-quadruplex anticoagulant 26-mer oligonucleotide with the sequence 5′-CGCCTAGGTTGGGTAGGGTGGTGGCG-3′ [22,23]. It has been established that this aptamer binds exosite I of thrombin with a high affinity through a highly complementary surface involving all three loops of the G-quadruplex module [24]. A current study has demonstrated almost equal anticoagulant activity for NU172 and TBA–GLE. Representative coagulation curves from experimental series that included these two samples showed virtually identical clotting profiles for both TBA–GLE and NU172 (Figure 3B). These results confirm our previous findings [13,25] that the formation of the extended recognition interface in the side chain is an efficient instrument for increasing the inhibitory and/or affinity characteristics of aptamers.

### 3.4. Molecular Dynamics of the Thrombin Complexes with GLE, SLE, SYE, and SYD Conjugates

**Figure 4:**
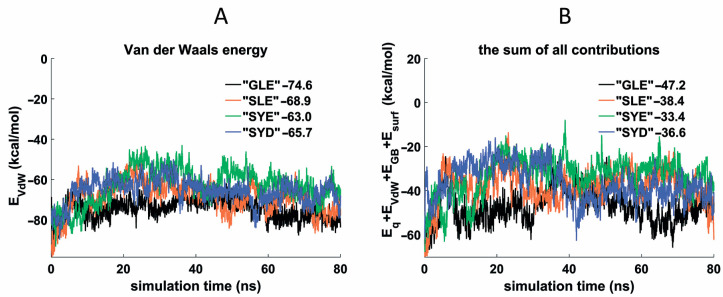
Van der Waals energy (**A**) and sum of all contributions (**B**) for thrombin complexes with TBA–GLE, TBA–SLE, TBA–SYE and TBA–SYD. The numerical data in the figures represent the mean values of the respective parameters.

Four TBA conjugates with GLE, SLE, SYE, and SYD peptide side chains at T3 were selected for molecular dynamics studies to compare Gly and Ser in position 17, Leu and Tyr in position 18, and Asp and Glu in position 19. Similarly to our previous study [13], we used the PDB structures 1HAO and 6Z8X to generate the starting models of the peptide conjugate complexes with thrombin. Following the established procedure, modified T3 residue was arranged in region B of the protein in proximity to Tyr76, and the total simulation time was set to 80 ns. The results of molecular dynamics generally confirmed the conclusions drawn from the coagulation experiments. The Van der Waals profiles and the total energy are the most representative in this respect (Figure 4). In the pair TBA–GLE and TBA–SLE, the former showed the lower average values of E_VdW_ and a lower sum for all contributions, thus supporting the preferable arrangement of Gly at position 17. Importantly, the complex of TBA–GLE with thrombin appeared to be the most stable structure in selected series of conjugates. With regard to the role of the aromatic vs. aliphatic amino acid in the middle of the peptide side chain, TBA–SLE formed a more stable complex with the protein than TBA–SYE. This finding also correlates with the results of coagulation studies, supporting an advantageous arrangement of hydrophobic aliphatic residues in this position. Finally, in the pair TBA–SYE and TBA–SYD, we compared an effect of the terminal amino acid on the potential energy of the complexes. Of the two amino acids, Asp and Glu, the former showed on average lower E_VdW_ and total energy values. Again, we should note a high correlation between the computational and experimental data. The energy contributions other than Van der Waals forces, as well as RMSD plots, are shown in Figure S4.

**Figure 5:**
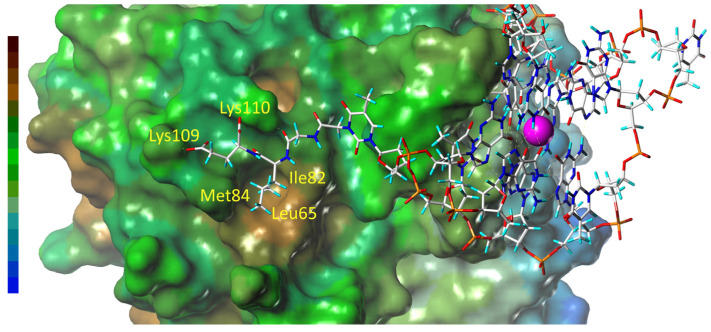
Snapshot of the thrombin complex with TBA–GLE at 57 ns (top view). Left bar represents the scale of hydrophobicity (the highest level is brown). The yellow labels in the figure refer to the key thrombin amino acids involved in the interaction with the peptide subunit.

Examining the TBA–GLE complex with thrombin in more detail revealed that the Leu18 residue of the tripeptide subunit allocates in the hydrophobic pocket of the thrombin in proximity to the protein amino acids Leu65, Ile82, and Met84 (Figure 5 and Figure S5). The instant interaction with these residues over the course of the simulation is confirmed by the virtually parallel accumulation of contact events between Leu18 and the three amino acids (Figure S6). On the other hand, the presence in the peptide subunit of the two terminal carboxylic groups was evidently beneficial in terms of electrostatic interaction since the peptide C-terminus is allocated in close proximity to Lys109 and Lys110 (Figure 5).

With regard to the TBA–peptide conjugate studies, the molecular dynamics simulations appeared to be a highly efficient tool in the verification of experimental data and elaborating general rules for the rational design or optimization of the peptide subunit. Indeed, our previous finding clearly referred to the base stacking between T3 and the peptide aromatic residues as a highly unfavorable event. Furthermore, it became clear that an additional negatively charged group at the C-terminus would be beneficial for the optimized structure. A current computational study has suggested the preferred allocation of Gly and Asp in the 17th and 19th positions of the tripeptide, respectively. Additionally, it confirmed that a nonaromatic hydrophobic amino acid is a preferable residue at position 18.

The molecular dynamics results confirmed that the GLE peptide represents the most favorable amino acid sequence and composition among the other variants examined. Nevertheless, the striking increase in anticoagulant activity upon a single replacement of Ser for Gly at position 17 raises the question of the nature of this effect. The CD spectra cannot be used to understand the origin of the effect since they only reflect the G-quartet organization. As for the molecular dynamics results, we could only see that GLE showed lower total energy compared to the other conjugates. In terms of the geometry of the peptide subunit, we can assume that both residues 16 and 17 play roles as linkers that grant optimal interactions between amino acids 18 and 19 and the protein. If this is the case, any functional substituent at residue 17 (such as the OH group in Ser) can potentially disrupt the optimal subunit arrangement due to possible interactions with the protein or DNA. However, these considerations are purely speculative and are not reliably supported by the available data.

## 4. Conclusions

A highly potent TBA–GLE aptamer anticoagulant was identified as a result of the optimization of the peptide subunit in a series of TBA–peptide conjugates. The lead structure is similar in its anticoagulant characteristics to the bimodular G-quadruplex aptamer NU172, an oligonucleotide that was the subject of Phase II clinical trials as an anticoagulant. The notably enhanced activity of TBA–GLE results from the presence of the optimized peptide side chain at the thymine base of the T3 residue, which functions as an extended aptamer recognition interface. The optimization strategy was based on the combination of the experimental screening of different TBA conjugates and computational methods, which, in turn, used detailed structural information available for TBA–thrombin complexes. This approach seems to be very promising for aptamer ligands with an established structure of their complexes with the corresponding protein target. Importantly, our approach represents a reasonable alternative to the expensive and time-consuming screening of a full-size conjugate library with all possible tripeptide subunits (8000 variants). Apart from the particular implication of TBA–peptide conjugates, it is noteworthy that improvements in affinity or inhibitory characteristics are an important addition to a wide range of biological functions that the peptide subunit may potentially perform in a covalent chimeric scaffold with a therapeutic oligonucleotide.

## Figures and Tables

**Table 1:** Characterization of TBA–peptide conjugates.

TBA Conjugate	M^calc^/M^obsvd^	Tm, °C ^1^	t_1/2_/t_1/2_^TBA 2^
SYN	5148.5/5151.5	50.9	1.56 ± 0.11
GYE	5133.5/5136.6	50.9	1.44 ± 0.26
SYE	5163.5/5171.3	50.1	1.72 ± 0.09
SWE	5186.6/5187.4	51.3	1.87 ± 0.19
GLE	5083.5/5084.3	50.8	5.98 ± 0.14
SYD	5149.5/5138.7	49.8	2.21 ± 0.12
SIE	5113.5/5116.5	50.7	3.15 ± 0.22
SLE	5113.5/5107.2	50.0	3.16 ± 0.32
SVE	5099.5/5098.2	50.3	2.63 ± 0.19
SPE	5097.5/5090.4	49.9	2.08 ± 0.28

^1^ in 100 mM KCl and 10 mM Na cacodylate (pH 7.4) at an oligonucleotide concentration of 5 μM; *T*_m_^TBA^ = 50.0 °C; Δ*T*_m_ = ± 0.5 °C; ^2^ relative clotting time ± SE. t_1/2_^NU172^/t_1/2_^TBA^ = 5.75 ± 0.08. In the clotting time measurements, the number of repeats was equal 2, except for SYN, SYE, SWE, and GLE (3); and TBA (6). The SE values were calculated as described elsewhere [20].

## Data Availability

Not applicable.

## Supplementary Materials

The following supporting information can be downloaded at: https://www.mdpi.com/article/10.3390/pharmaceutics15020604/s1. Figure S1. Modified thymidine phosphoramidite with p-nitrophenyl activated carboxylic function at N3 of pyrimidine nucleobase. Figure S2. Analysis of TBA-peptide conjugates by electrophoresis in denaturing polyacrylamide gel (19:1, 7M urea, 1×TBE). Detection by UV shadowing. Figure S3. MALDI mass spectra of TBA-peptide conjugate. Figure S4. Energy contributions and RMSD plots for thrombin complexes with TBA-GLE, TBA-SLE, TBA-SYE, and TBA-SYD: the sum of electrostatic and polar solvate energy (A); non-polar solvate energy (B); RMSD values for peptide subunits (C), G-quadruplex modules (D), and conjugates (E). The numerical data in the figures represent the mean values of the respective parameters. Figure S5. Snapshot of the thrombin complex with TBA–GLE at 57 ns (side view). Left bar represents the scale of hydrophobicity (the highest level is brown). Figure S6. Accumulation of contact events between Leu18 and the protein amino acids Leu65, Ile82, and Met84 in the complex thrombin-(TBA-GLE).
