# Supplementary material for: The Regioselective Conjugation of the 15-nt Thrombin Aptamer with an Optimized Tripeptide Sequence Greatly Increases the Anticoagulant Activity of the Aptamer
Source: Pharmaceutics. 2023 Feb 10;15(2):604. doi: 10.3390/pharmaceutics15020604 (PMC9967020; doi:10.3390/pharmaceutics15020604)
Supplement: Supplementary file 1 [file pharmaceutics-15-00604-s001.zip › pharmaceutics-2107042-supplementary.pdf]

Supplementary Materials

The regioselective conjugation of the 15-nt thrombin aptamer with an optimized tripeptide sequence greatly increases the anticoagulant activity of the aptamer

Irina V. Varizhuk[1], Vladimir B. Tsvetkov[2,3], Ilya Yu. Toropygin[4], Andrey A. Stomakhin[1], Natalia A. Kolganova[1], Sergei A. Surzhikov[1], Edward N. Timofeev[1]*

[1]Engelhardt Institute of Molecular Biology, Russian Academy of Sciences, 119991 Moscow, Russia
[2]Federal Research and Clinical Center of Physical-Chemical Medicine, 119435 Moscow, Russia
[3]Institute of Biodesign and Complex System Modeling, Sechenov First Moscow State Medical University, 119146 Moscow, Russia
[4]Department of Proteomics, V.N. Orekhovich Research Institute of Biomedical Chemistry, Russian Academy of Medical Sciences, Moscow 119832, Russia
*Correspondence: edward@eimb.ru

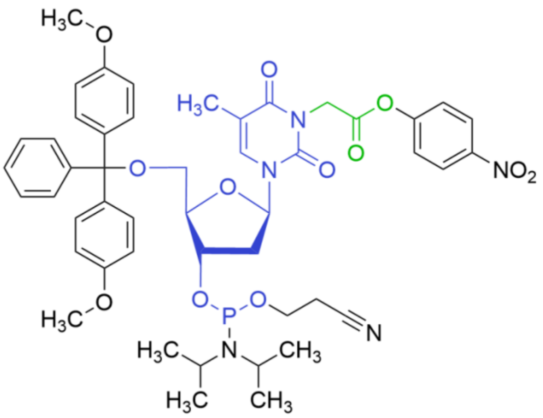

Figure S1. Modified thymidine phosphoramidite with p-nitrophenyl activated carboxylic function at N3 of pyrimidine nucleobase.

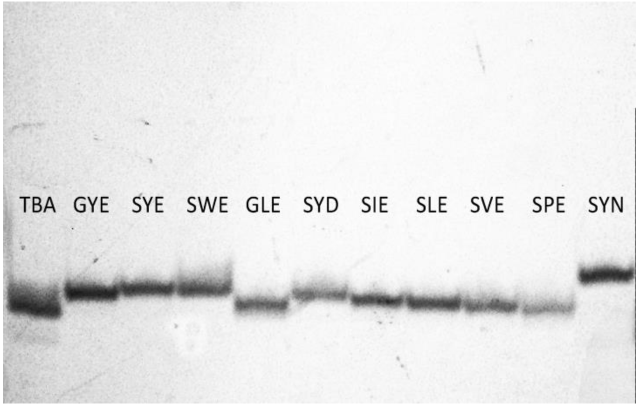

Figure S2. Analysis of TBA-peptide conjugates by electrophoresis in denaturing polyacrylamide gel (19:1, 7M urea, 1×TBE). Detection by UV shadowing.

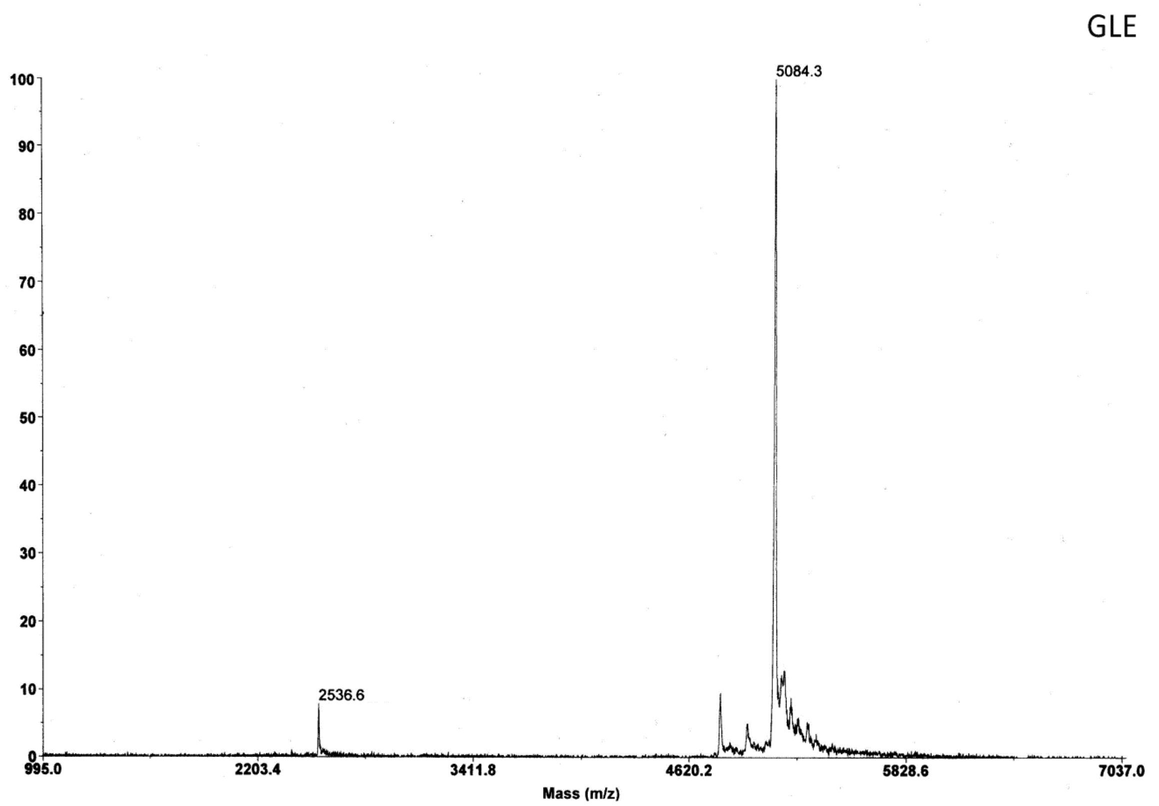

GLE
5084.3
2536.6
100
90
80
70
60
50
40
30
20
10
0
995.0
2203.4
3411.8
4620.2
5828.6
7037.0
Mass (m/z)

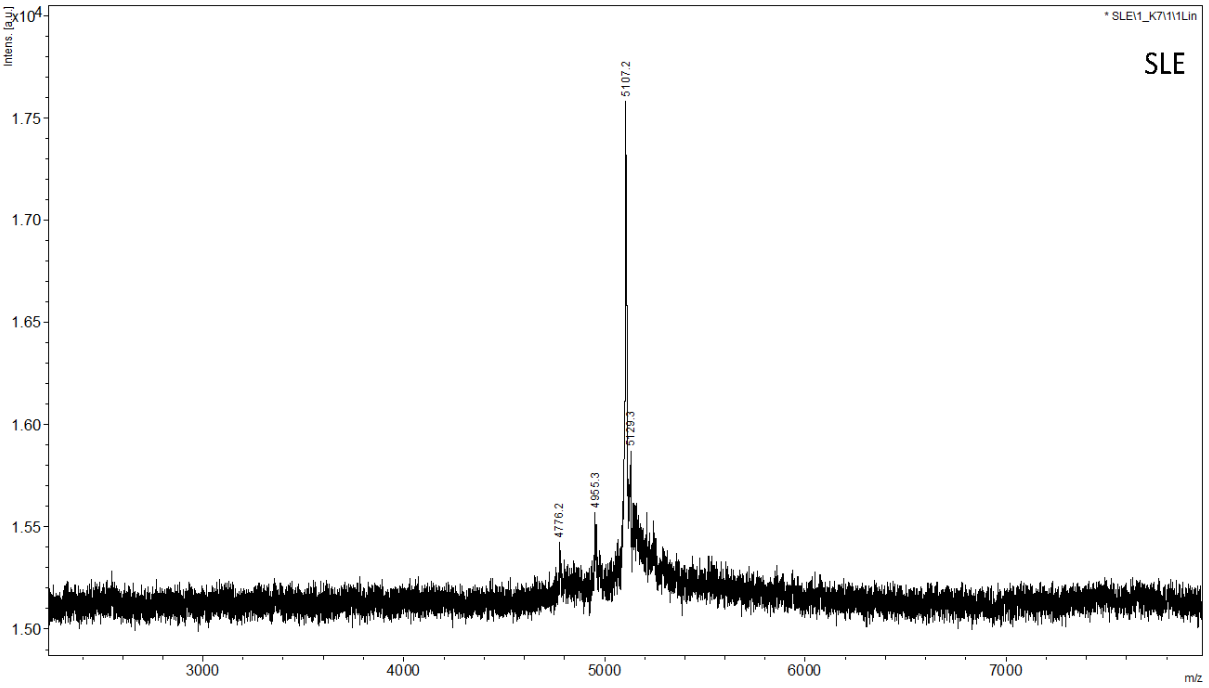

x10^4
Intens. [a.u.]
* SLE\1_K7\1\1Lin
SLE
5107.2
5129.3
4955.3
4776.2
1.75
1.70
1.65
1.60
1.55
1.50
3000
4000
5000
6000
7000
m/z

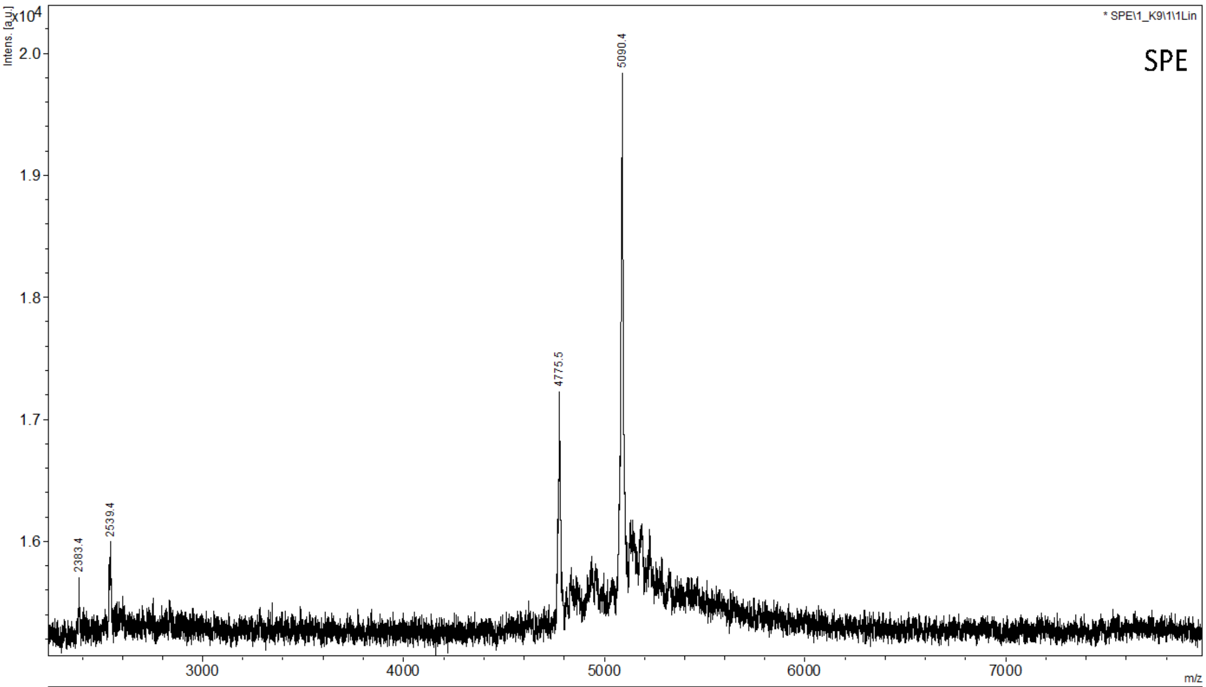
SPE
* SPE\1_K9\1\1Lin
Intens. [a.u.]
x10^4
2.0
1.9
1.8
1.7
1.6
5090.4
4775.5
2539.4
2383.4
3000
4000
5000
6000
7000
m/z

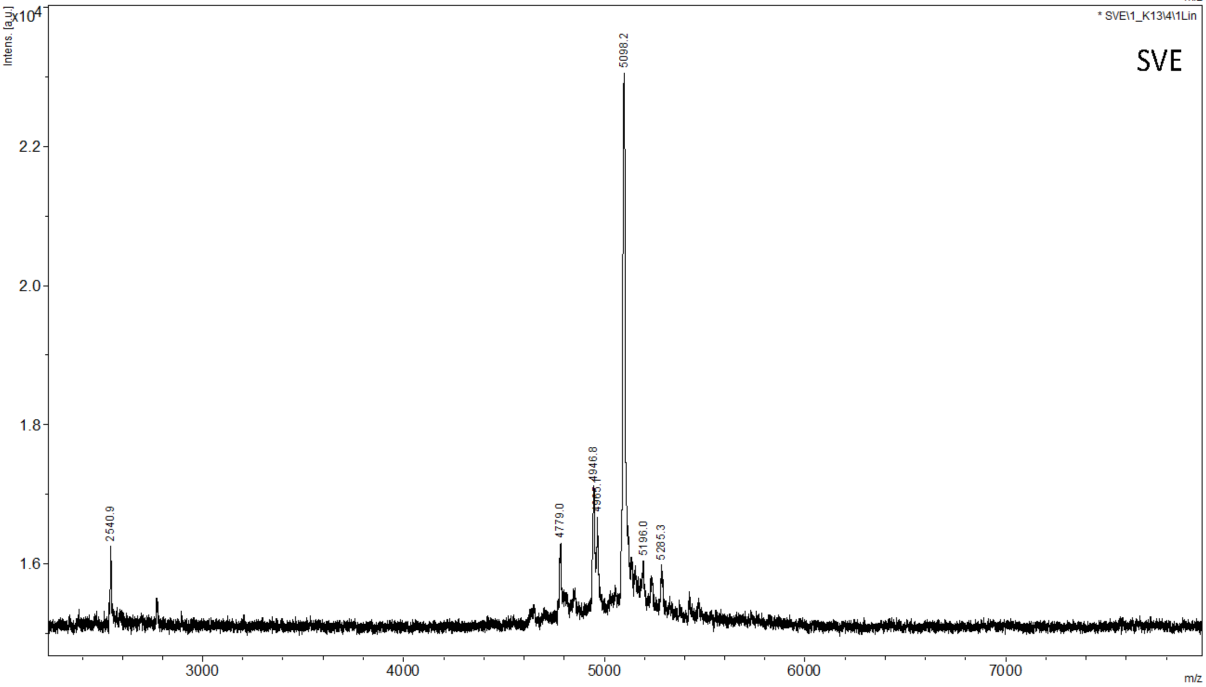
SVE
* SVE\1_K13\4\1Lin
Intens. [a.u.]
x10^4
2.2
2.0
1.8
1.6
5098.2
4946.8
4779.0
2540.9
5196.0
5285.3
3000
4000
5000
6000
7000
m/z

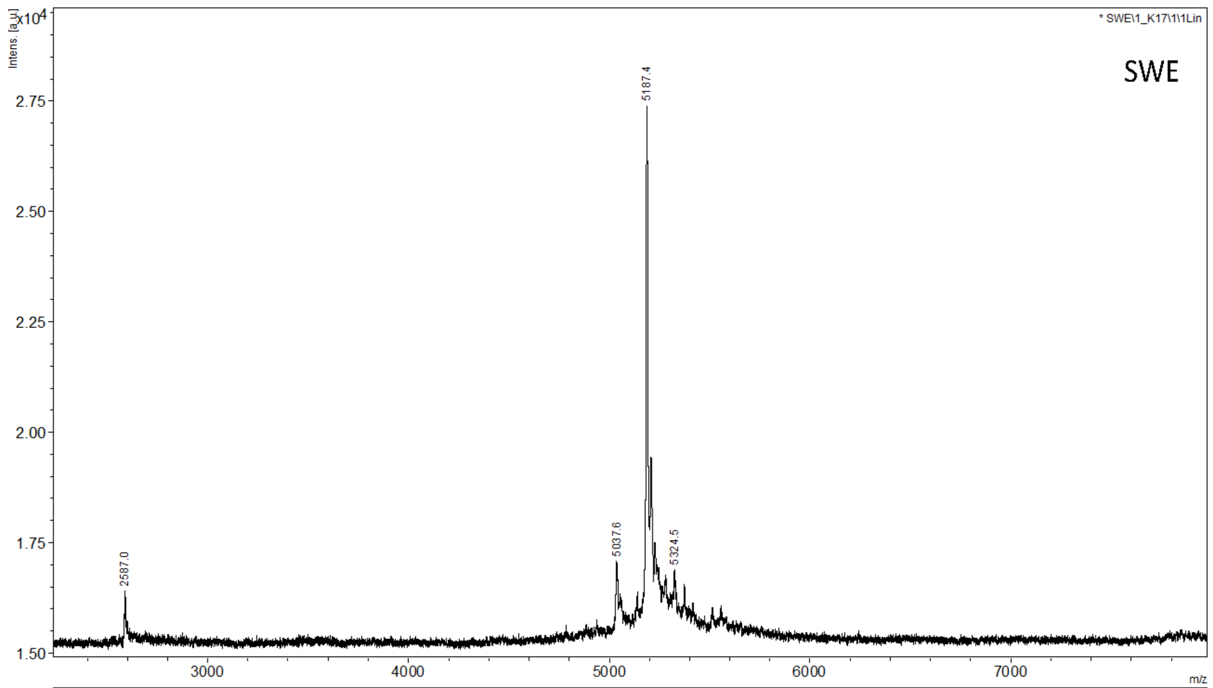
* SWE\1_K17\1\1Lin
SWE
Intens. [a.u.]
x10^4
5187.4
5037.6
5324.5
2587.0
2.75
2.50
2.25
2.00
1.75
1.50
3000
4000
5000
6000
7000
m/z

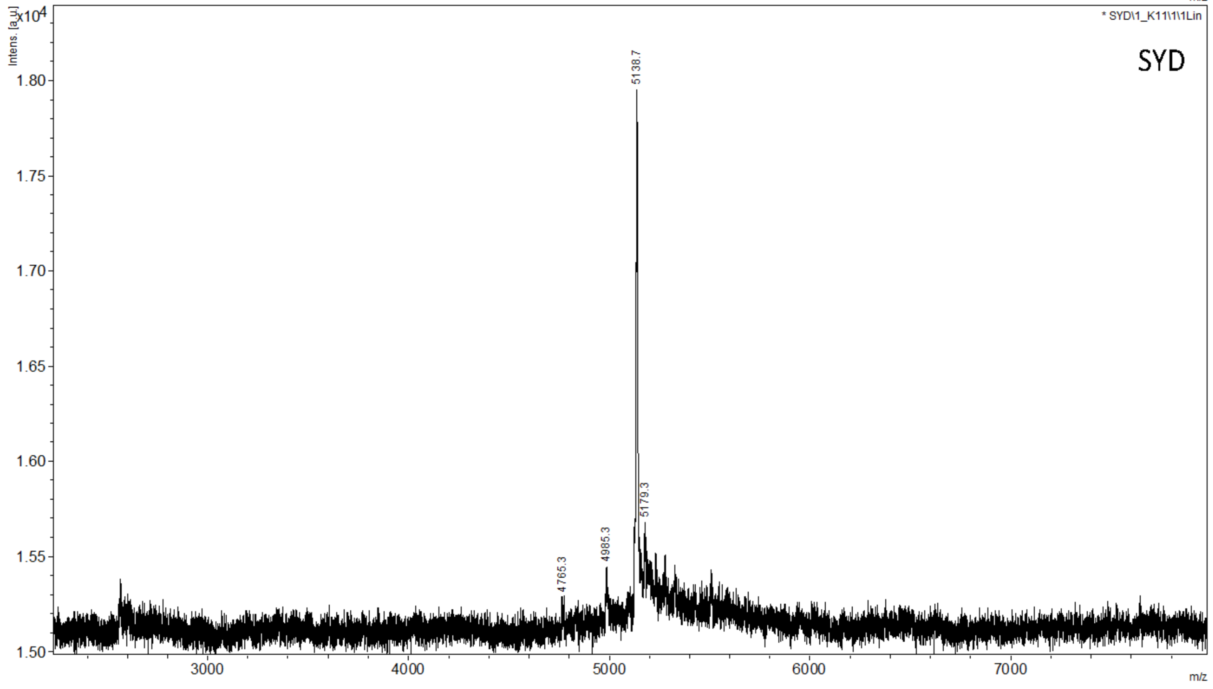
* SYD\1_K11\1\1Lin
SYD
Intens. [a.u.]
x10^4
5138.7
5179.3
4985.3
4765.3
1.80
1.75
1.70
1.65
1.60
1.55
1.50
3000
4000
5000
6000
7000
m/z

SYE

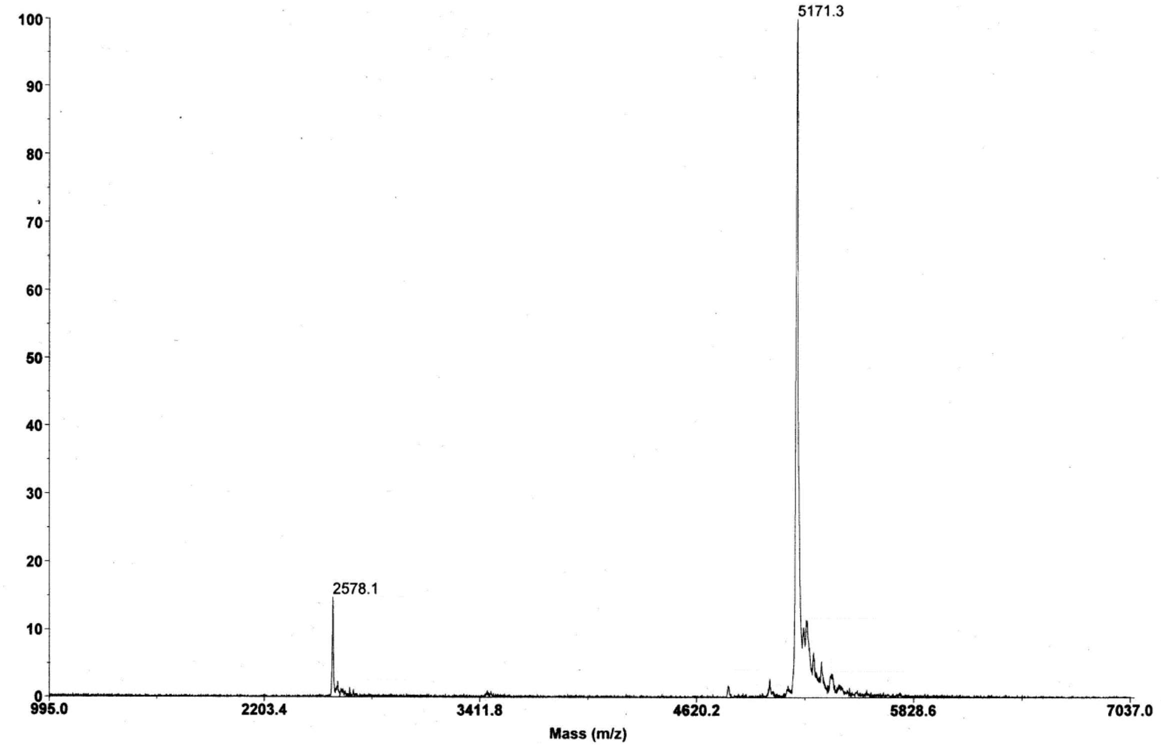
5171.3
2578.1
100
90
80
70
60
50
40
30
20
10
0
995.0
2203.4
3411.8
4620.2
5828.6
7037.0
Mass (m/z)

SYN

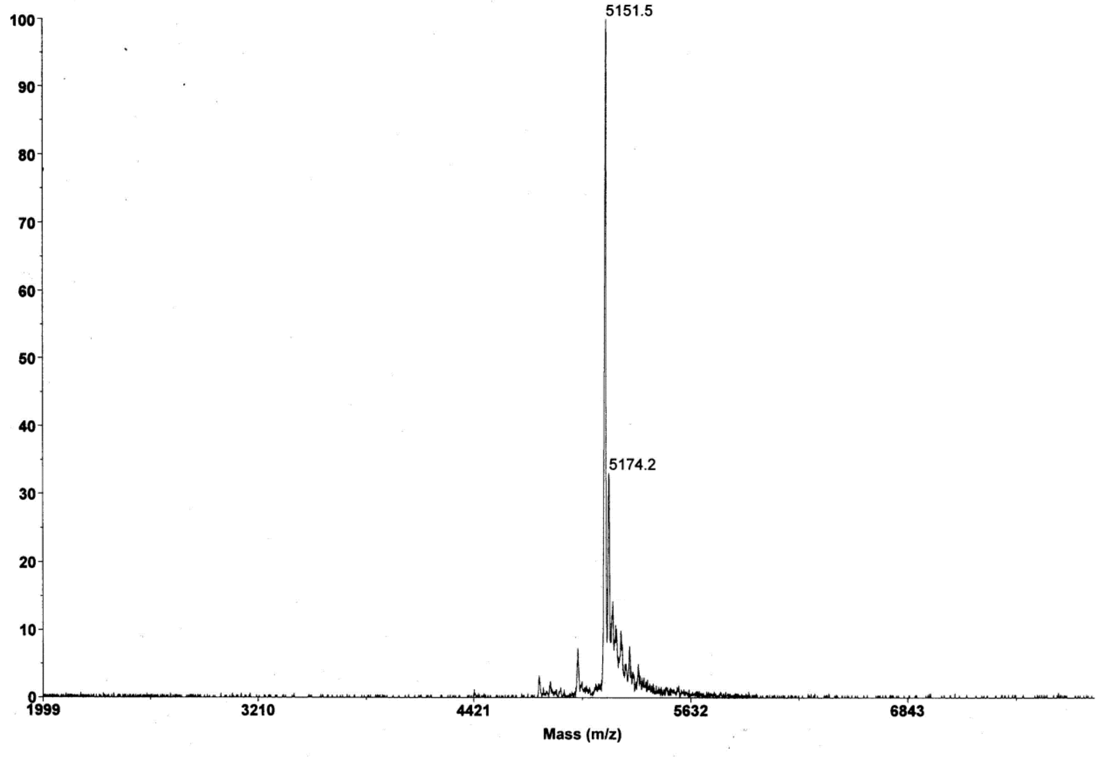
5151.5
5174.2
100
90
80
70
60
50
40
30
20
10
0
1999
3210
4421
5632
6843
Mass (m/z)

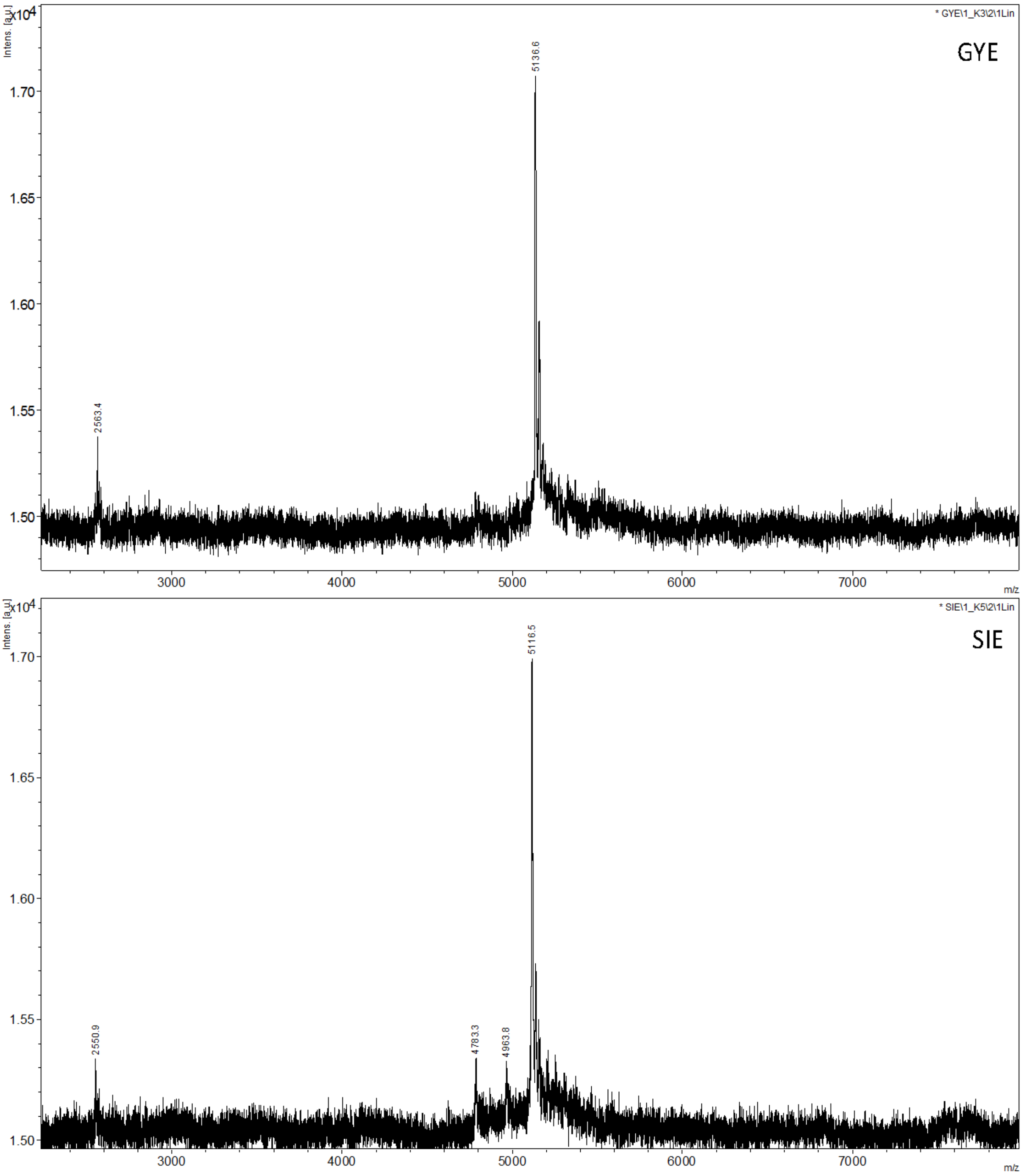

Figure S3. MALDI mass spectra of TBA-peptide conjugates.

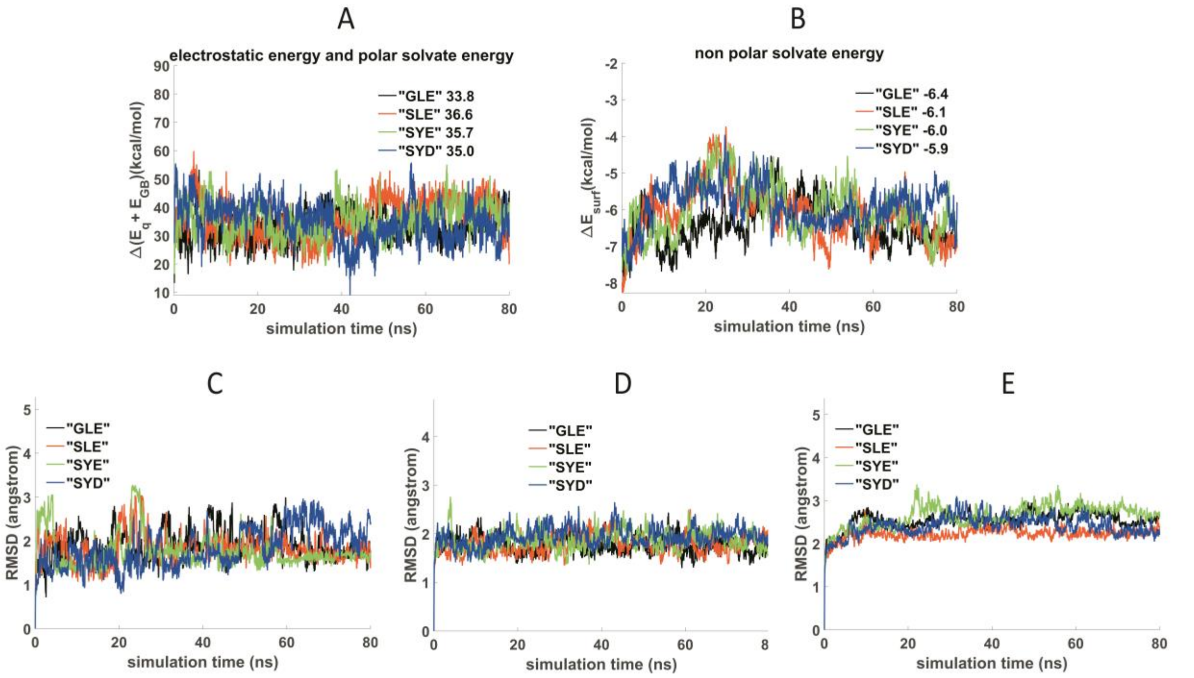

Figure S4. Energy contributions and RMSD plots for thrombin complexes with TBA-GLE, TBA-SLE, TBA-SYE, and TBA-SYD: the sum of electrostatic and polar solvate energy (A); non-polar solvate energy (B); RMSD values for peptide subunits (C), G-quadruplex modules (D), and conjugates (E). The numerical data in the figures represent the mean values of the respective parameters.

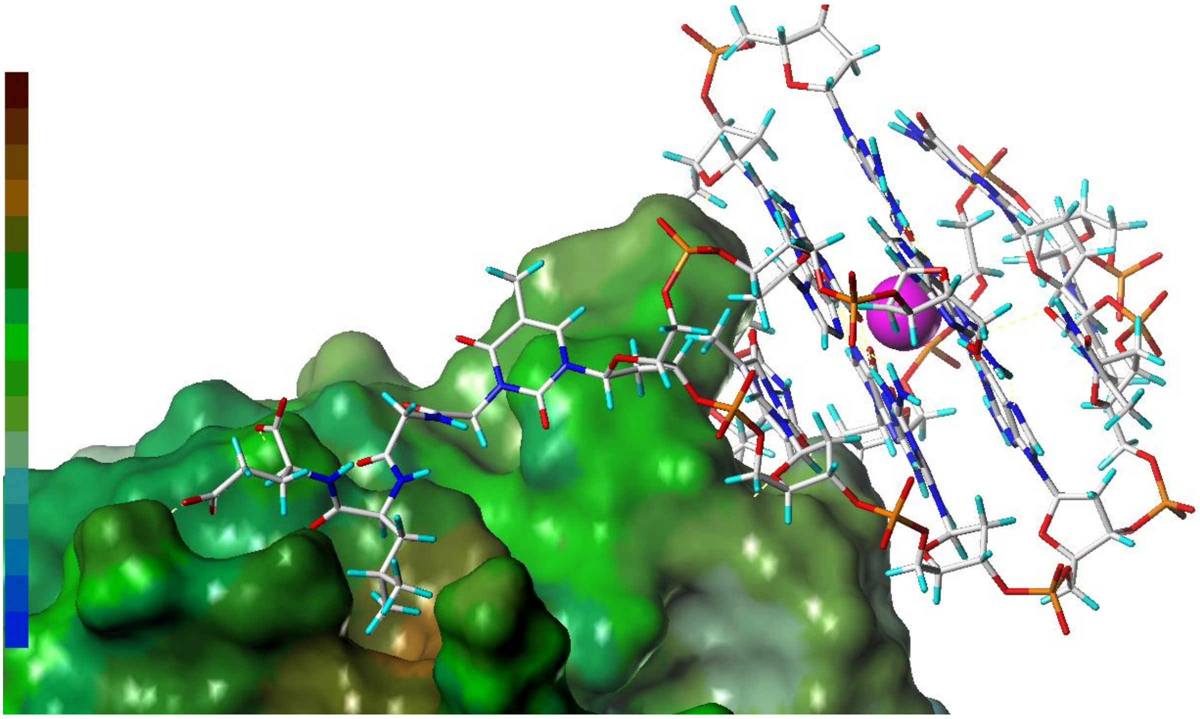

Figure S5. Snapshot of the thrombin complex with TBA–GLE at 57 ns (side view). Left bar represents the scale of hydrophobicity (the highest level is brown).

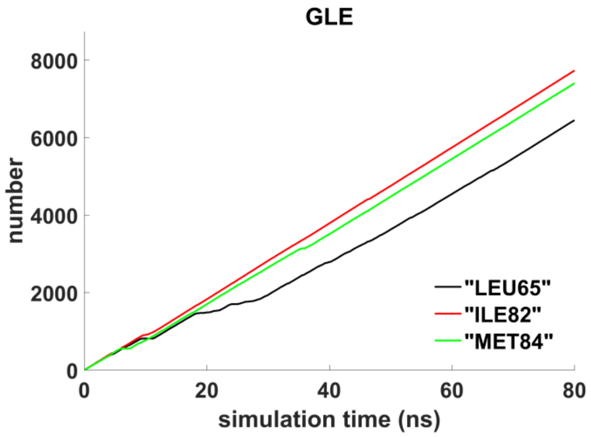

Figure S6. Accumulation of contact events between Leu18 and the protein amino acids Leu65, Ile82, and Met84 in the complex thrombin-(TBA-GLE).
